# Are baseline ultrasound and mammographic features associated with rates of pathological completes response in patients receiving neoadjuvant chemotherapy for breast cancer?

**DOI:** 10.1186/s40644-019-0251-3

**Published:** 2019-10-21

**Authors:** Sarah L. Savaridas, Yee Ting Sim, Sarah J. Vinnicombe, Colin A. Purdie, Alastair M. Thompson, Andy Evans

**Affiliations:** 1 University of Dundee, Dundee, UK; 2 Ninewells Hospital, Dundee, UK; 3 Thirlestaine Breast Centre, Cheltenham, UK; 4 Baylor College of Medicine, Houston, USA

**Keywords:** Breast carcinoma, Neoadjuvant chemotherapy, Pathological complete response, Mammography, Ultrasound

## Abstract

**Background:**

Increasing numbers of breast cancer patients receive neoadjuvant chemotherapy (NACT). We seek to investigate whether baseline mammographic and ultrasound features are associated with complete pathological response (pCR) after NACT.

**Methods:**

A database of NACT patients was reviewed. Baseline imaging parameters assessed were ultrasound: posterior effect; echo pattern; margin and lesion diameter; mammography: spiculation and microcalcification. Core biopsy grade and immunophenotype were documented. Data were analysed for the whole study group and by immunophenotype.

**Results:**

Of the 222 cancers, 83 (37%) were triple negative (TN), 61 (27%) ER positive/HER-2 negative and 78 (35%) HER-2 positive. A pCR occurred in 46 of 222 cancers (21%). For the whole group, response was associated with high core biopsy grade (grade 3 vs. grade 1 or 2) (26% vs. 9%, *p* = 0.0044), absence of posterior shadowing on ultrasound (26% vs. 10%, *p* <  0.001) and the absence of mammographic spiculation (26 vs. 6%, *p* <  0.001). Within the HER-2 positive group; the absence of shadowing and spiculation remained highly associated with pCR, in addition to small ultrasound size (AUC = 0.71, *p* < 0.001) and the absence of microcalcification (39% vs. 21%, *p* < 0.02). On multivariable analysis absence of spiculation and core grade remained significant for the whole cohort, size and absence of spiculation remained significant for HER-2 positive tumours. No feature predicted pCR in TN tumours.

**Conclusion:**

A pCR is less likely when there is mammographic spiculation. Small ultrasound size is associated with pCR in HER-2 positive tumours. These findings may be helpful when discussing NACT and surgical options with patients.

**Trial registration:**

UK Clinical Trials Gateway: registration number 16712.

## Background

Increasing numbers of breast cancer patients are now receiving neoadjuvant chemotherapy (NACT). Whilst in some patients this results in down-staging of an initially inoperable tumour or reduces the extent of surgery required in the breast and axilla, others derive little, if any, benefit from NACT yet experience the associated morbidity of treatments. Patients might choose to have chemotherapy post operatively if they were aware that NACT was unlikely to result in a sufficiently good response to alter the surgical plan. Currently the only predictors of response used routinely are the immunophenotype of the tumour and core biopsy tumour grade. However, large variations are seen in chemo(in)sensitivity within immunophenotypes and tumours of the same grade. Therefore, increasing the physician’s ability to predict at baseline the response to NACT would be helpful for patient selection.

Various genetic and immunohistochemical tumour factors have been proposed to aid prediction of response, however these remain imperfect and are not in routine use [1–3]. There are a few published studies assessing features of baseline MRI scans, demonstrating that well-defined and round/oval tumours, absence of intra-tumoural high T2 signal intensity and absence of peri-tumoural oedema correlate with better response to NACT [4–6]. Evidence pertaining to baseline ultrasound and mammography, however, remains scant. Two recent papers considered triple negative cancers alone; one study of 328 patients found the presence of microcalcification on the initial mammogram was significantly associated with residual disease (presumed to include in-situ disease) [7]. A further study comparing tumours that completely responded to tumours with residual in-situ disease following NACT found that the absence of mammographic microcalcification, round shape and posterior enhancement on baseline ultrasound were significantly more common amongst tumours which demonstrated pCR with no residual in-situ disease [8]. However, in neither study, were these findings significant on multivariable analysis. To our knowledge there is no published evidence regarding baseline mammographic or ultrasound imaging features and response prediction in other breast cancer immunophenotypes. Mammography (MMG) and ultrasound (US) are almost universally performed at diagnosis, prior to treatment decisions and the commencement of NACT. Thus, if it were possible to identify baseline imaging features associated with treatment response this would provide an inexpensive and readily available guide for the treating team. Our aim was therefore to identify associations between baseline US features, mammographic characteristics and other tumour parameters routinely available pre-treatment and pCR in women receiving NACT both as a whole and according to immunophenotype.

## Methods

This was a retrospective review of data collected as part of an ethically-approved prospective breast cancer imaging study (REC no. 14/ES/0047). Women over the age of 18 years with invasive breast cancer receiving NACT were included after written informed consent. All patients were metastasis free at the start of treatment. Baseline ultrasound (US) and mammography (MMG) was performed at diagnosis. Ultrasound was performed on SuperSonic Imagine (SuperSonic, Aix-en-Provence France). Mammograms were performed on either Hologic Selina Dimensions (Hologic, Bedford, Massachusetts) or Seimans Mammomat Inspiration (Seimans-Healthineers, Erlangen, Germany) Imaging was assessed by independently by two experienced breast radiologists who were blinded to the treatment outcomes. US imaging features documented included posterior effect (shadowing, no effect or enhancement), echo pattern (hypoechoic, hyperechoic or heterogeneous), maximum lesion size and whether the lesion had circumscribed margins.

The MMG features documented were the presence or absence of spiculation (the presence of either distortion or a spiculate mass) and tumour-associated microcalcification. Estimated tumour grade and tumour immunophenotype as assessed on diagnostic core biopsy were documented as these factors are available at baseline assessment. The HER-2 positive group included those with ER and/or PR positivity, therefore the ER positive group does not include ER positive tumours which were also HER-2 positive. Complete pathological response (pCR) was defined as the absence of invasive disease in both the breast and the axilla in the final post NACT surgical specimen. Lesions with residual DCIS but no invasion were therefore counted as a pCR.

Analysis was performed both for the whole study group and by tumour immunophenotype. Owing to small numbers in some subsets, data were reviewed and grouped for statistical analysis. For US posterior effect, the results were similar for enhancement and no effect thus the data were grouped into shadowing vs non-shadowing. Similarly, the results for hyperechoic and heterogeneous lesional echo pattern were similar and therefore echo pattern was analysed as hypoechoic vs non-hypoechoic. Univariate analysis of categorical data was performed using chi square, and continuous data using ROC analysis. Multivariate analysis was performed using a logistic regression model. Medcalc software was used for statistical analysis.

## Results

Two hundred twenty-two women were included with an average age of 52.0 years (range: 24–79 years). Age was not associated with pCR (area under the curve for whole group 0.55, *p* = 0.3). Of the total cancers, 83 (37%) were triple negative (TN), 61 (27%) ER positive/HER-2 negative and 78 (35%) HER-2 positive.

Complete pathological response was achieved in 46 (21%) patients. Within the subgroups, similar rates of pCR were seen in the TN and HER-2 positive groups; (19/83) 22.9% and (23/78) 29.4% respectively. By contrast, only four (6.6%) patients in the ER positive HER-2 negative group achieved a pCR rendering subgroup analysis of these women impracticable.

The majority (154, 69%) of tumours were estimated grade 3 on core biopsy, 66 (30%) were grade 2 and 2 (1%) grade 1. Owing to the very low numbers in the grade 1 group, the data were combined with the grade 2 group for further analysis. Complete response was achieved significantly more frequently with core biopsy grade 3 tumours than in grade 1 & 2 tumours; 26% vs 9% respectively (*p* = 0.0044). However, grade did not remain significant in subgroup analysis of TN and HER-2 positive cancers.

**Fig. 1:**
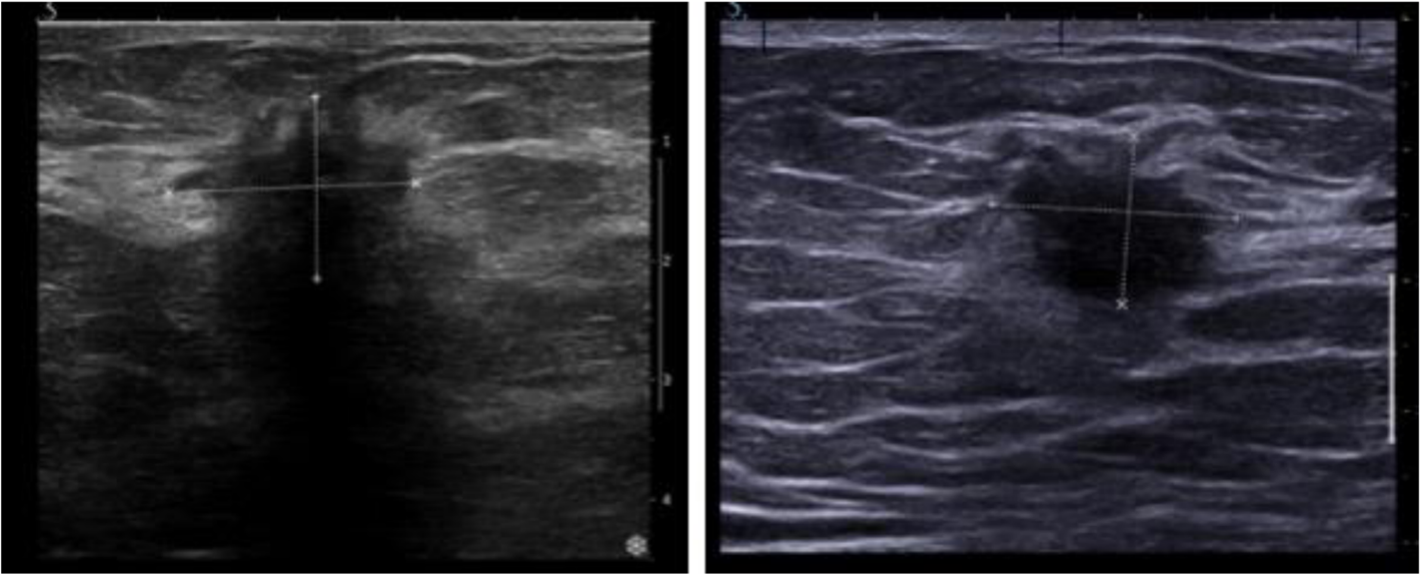
**a** Illustrates the appearance of posterior shadowing on ultrasound of an invasive carcinoma. The patient died from metastatic breast cancer, less than 2 years after diagnosis. **b** Shows a grade 3 invasive ductal carcinoma, the patient remains metastasis free 6 years post diagnosis

**Fig. 2:**
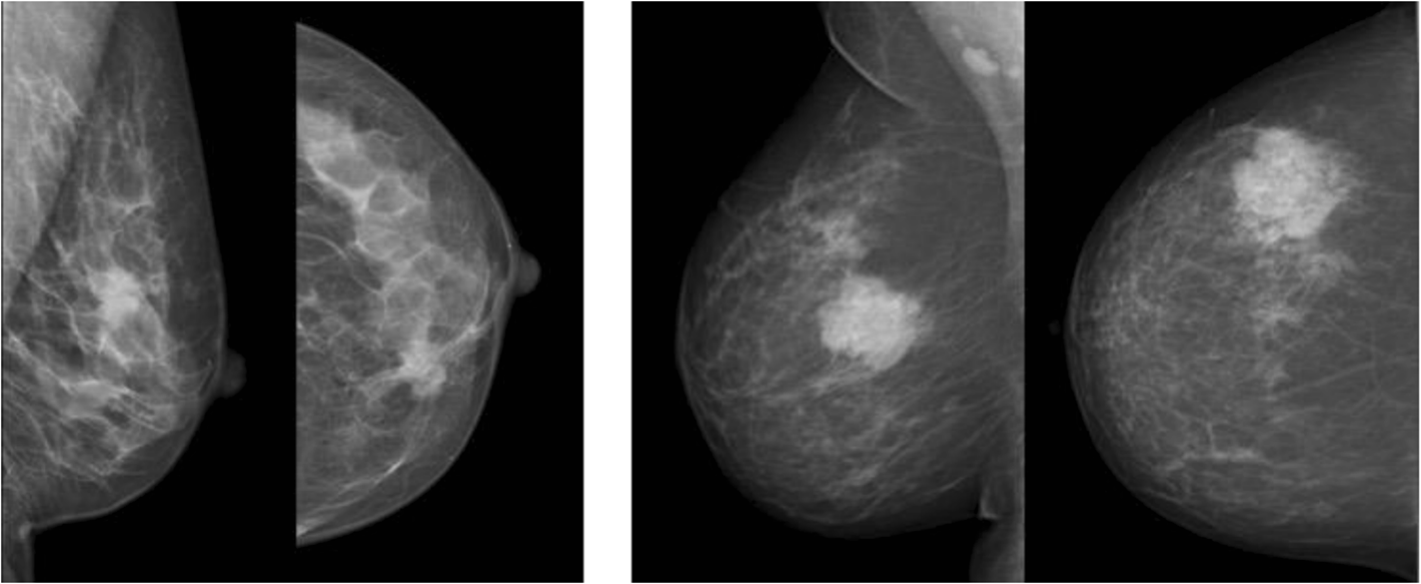
**a** Left MLO and CC views spiculate mass due to a grade 2 invasive ductal carcinoma. **b** Right MLO and CC views illustrating a non-spiculate mass, this was a grade 3 invasive ductal carcinoma

One patient was unable to have a MMG at diagnosis, while all patients underwent US. The combined results of the two readers are considered, thus there are 442 MMG and 444 US interpretations. On univariate analysis of the whole group data absence of posterior shadowing on ultrasound (76 of 291; 26% vs. 16 of 153; 10%, *p* < 0.001) was associated with pCR. The presence of distal enhancement on ultrasound was not associated with pCR. Figure 1a illustrates posterior shadowing, this is the ultrasound imaging of a 56-year-old patient, who died 2 years after diagnosis. By contrast Fig. 1b illustrates a tumour demonstrating no posterior effect, this tumour had a complete response to NACT and the patient remains metastasis free 6 years after diagnosis. The absence of spiculation on mammography (85 of 321; 27% vs. 7 of 121; 6%, *p* < 0.001) was also significantly associated with pCR (see Table 1). Examples of spiculated and non-spiculated masses are given in Fig. 2.

**Table 1:** Rates of pathological complete response according to selected baseline radiological feature for all tumours

	pCR	Non pCR	% pCR	*p* value
No shadowing	76	215	26.1	
Shadowing	16	137	10.5	**< 0.001**
Hypoechoic	67	241	21.8	
Non-hypoechoic	25	111	18.4	0.42
Circumscribed	26	72	26.5	
Not circumscribed	66	280	19.1	0.11
Calcification	25	126	16.6	
No calcification	67	224	23.0	0.11
Spiculation	7	114	5.8	
No spiculation	85	236	26.5	**< 0.001**
Size	AUC 0.588			0.059

The presence of a circumscribed margin (26 of 98; 27% vs. 66 of 346; 19%, *p* = 0.11), the echo pattern (hypoechoic 67 of 308; 22% vs. non-hypoechoic 25 of 136; 18%, *p* = 0.4), size (area under curve 0.059; *p* = 0.059) on US, and mammographic microcalcification (25 of 151; 17% vs. 67 of 291; 23%, *p* = 0.11) on mammography were not significantly associated with response to NACT.

The absence of shadowing and spiculation remained highly significant predictors of pCR on univariate analysis of the HER-2 positive group, in addition to small ultrasound size (AUC = 0.713, *p* < 0.001) (Fig. 2) and absence of microcalcification (29 of 74; 39% vs. 17 of 80; 21% *p* < 0.02) (Table 2). By contrast, no feature (including US size) was significantly associated with response in TN tumours (Table 3).

**Table 2:** Rates of pathological complete response according to selected baseline radiological feature for HER-2 positive tumours

	pCR	Non pCR	% pCR	*p* value
No shadowing	37	59	38.5	
Shadowing	9	51	15.0	**0.002**
Hypoechoic	37	76	32.7	
Non-hypoechoic	9	34	20.9	0.15
circumscribed	10	12	45.4	
Not circumscribed	36	98	26.9	0.08
calcification	17	63	21.3	
No calcification	29	45	39.2	**0.02**
spiculation	2	37	5.1	
No spiculation	44	71	38.3	**< 0.001**
ER + ve	12	40		
ER -ve	11	15		0.08
Size	AUC = 0.713			**< 0.001**

**Table 3:** Rates of pathological complete response according to selected baseline radiological feature for TN tumours

	pCR	Non pCR	% pCR	*p* value
No shadowing	35	107	24.6	
Shadowing	3	21	12.5	0.19
Hypoechoic	24	94	20.3	
Non-hypoechoic	14	34	29.2	0.22
circumscribed	16	56	22.2	
Not circumscribed	22	72	23.4	0.86
calcification	8	28	22.2	
No calcification	30	100	23.1	1
spiculation	2	12	14.3	
No spiculation	36	116	23.7	0.42
Size	AUC 0.525			0.75

For the entire cohort, absence of spiculation and core grade remained significant on multivariate analysis (Table 4). Within the HER-2 positive group; size and absence of spiculation remained significant (Table 5).

**Table 4:** Final multivariate model for the whole cohort

	Odds Ratio	95% CI	*p*
Shadowing	1.66	0.88–3.13	0.12
Spiculation	3.70	1.57–8.74	**0.003**
Core grade	0.38	0.20–0.73	**0.004**

**Table 5:** Final multivariate model for HER-2 positive tumours

	Odds Ratio	95% CI	*p*
Shadowing	2.42	0.95–6.20	0.07
Calcification	1.35	0.59–3.11	0.48
Spiculation	9.00	1.90–42.6	**0.006**
Size	1.10	1.04–1.16	**0.001**
Core grade	0.93	0.28–3.10	0.90

## Discussion

Neoadjuvant chemotherapy is used to treat increasing numbers of women with breast cancer, especially those with locally advanced disease. Selecting patients most likely to derive benefit from treatment is essential. We have demonstrated that baseline imaging features on both US and mammography are associated with pCR rates, and therefore may be a useful tool in identifying patients who will respond well. Key imaging features included absence of posterior shadowing on ultrasound and the absence of spiculation on mammography for both the whole group and the HER-2 positive sub-group. Along with core biopsy grade, absence of spiculation remained significantly correlated with pCR on multivariate analysis of whole group data. In the HER-2 positive subgroup, mammographic spiculation and large ultrasound size were significantly associated with residual disease on multivariate analysis. Whilst there was a trend towards an association of absence of shadowing and absence of spiculation with pCR in the TN group, these were not statistically significant.

### Immunophenotype and grade

It is widely accepted that the histological subtype and grade of tumour affects the likelihood of response to NACT. In agreement with previous published work, we found that pCR occurred most commonly in high grade tumours [9–11]. This association remained significant on multivariate analysis of whole group data but was lost on subgroup analysis, perhaps due to the relatively small numbers. In a meta-analysis of 30 studies with approximately 1000 subjects, Houssami et al demonstrated an independent association between breast cancer subtype and pCR, with odds of pCR highest in the HER-2 positive and TN subtypes [12]. Our findings are congruent with their study, which showed a pooled pCR of 19% and pCR for the subtypes: HER-2+ 29%, TN 31% and HR+/HER-2- 9% respectively. The lower pCR rates amongst ER positive tumours may be partly due to the correlation between ER-positivity and lower tumour grade [9].

### Gene expression and radiological correlates

Several molecular assays have been developed to predict likelihood of disease recurrence and/or response to neoadjuvant chemotherapy, including Oncotype DX and MammaPrint/BluePrint [1]. OncotypeDX is based on an expression profile of 21 genes and generates a predicted recurrence score (ODRS). A high RS has shown potential for predicting pCR in patients with HER-2 negative tumours [3]. Interestingly, higher ODRS have demonstrated a correlation with an oval mass at mammography and US posterior enhancement [13].

### Spiculation

Spiculated margins at mammography are present significantly more frequently in low grade tumours [14, 15]. It has been postulated that this is due to the desmoplastic reaction more frequently provoked by low grade tumours [15] and ER positive (luminal A type) tumours [16]. These tumour subtypes also tend to respond less well to NACT. By contrast, triple negative tumours, which more frequently respond to NACT are predominantly high grade and infrequently spiculate [17, 18]. Furthermore, it has been demonstrated that a pCR is more likely in lesions that are well defined, oval or round than in those that are diffuse or irregular [5, 6]. Therefore the correlation we have identified between the presence of spiculation and lower rates of pCR is perhaps not surprising.

### Absence of posterior shadowing

It has previously been demonstrated that low grade tumours tend to produce acoustic shadowing, whilst 36% of high-grade tumours demonstrate acoustic enhancement on ultrasound examination [14]. Given that it is widely accepted that low grade tumours are less likely to respond to NACT, [9–11] this may explain the correlation with absence of posterior shadowing and pCR in our study. This correlation is lost on multivariate analysis. This may be explained by the association between tumour grade and posterior acoustic shadowing.

### Size

Although not specific to ultrasound, there is evidence that smaller tumours (defined as T1 or ≤ 2 cm) are significantly more likely to achieve a complete response [9]. A recent study looked at correlations between initial tumour size and pCR following NACT. Tumour size was taken as the largest dimension on pre-treatment imaging (mammography, ultrasound, MRI, PET-CT, PET-mammography or CT). On univariate analysis, the probability of pCR significantly decreased with increasing tumour size in the basal and HER-2 subgroups, however in multivariate logistic regression analysis this correlation was lost for all subgroups [19]. This is at slight variance to our findings for whole group and HER-2 positive tumours, with ultrasound size correlating with pCR on univariate analysis and remaining significant in multivariate analysis of HER-2 positive group. Whilst the basal status of the tumours in our series is unknown, there is at least 70% concordance between triple negative and basal tumours [20]. It is of note that by contrast we found no hint of a relationship between US size and response in the TN sub-group. Other studies have shown little or no relationship between size and survival in TN breast cancer, particularly in those expressing basal cytokeratins [21]. This suggests that innate characteristics of the tumour are more important than size and stage in determining the outcome of TN tumours.

### Microcalcifications

On univariate analysis, in the HER-2 positive subgroup, microcalcification was a negative predictor for pCR, although this did not remain significant on multivariate analysis. This is consistent with existing evidence that comedo-type, casting and pleomorphic microcalcifications on initial mammography are associated with poorer prognosis [22–26]. Interestingly, in contrast to two recent studies, we found no correlation between microcalcification and poorer response to NACT in the TN cancers [7, 8]. This may be due to the differing definitions of complete response; whilst in our study pCR is considered the absence of invasive disease the previous studies considered complete response to be the absence of both invasive and in-situ disease. Microcalcification is commonly associated with the presence of ductal carcinoma in situ (DCIS). The lack of correlation may also be related to the relatively small proportion of TN demonstrating microcalcification on mammographic interpretation; 36/166, 22%.

### Limitations

This was a single centre study with relatively low numbers, particularly in certain immunophenotypic subgroups. The rate of pCR is also low, probably because NACT is only given in our centre for large tumours and/or node positive patients. The findings of this study may therefore not reflect what is found when NACT is given to smaller tumours on the basis of immunophenotype. Some of the factors which lost significance on multivariate analysis may be of interest as the loss of significance could reflect small numbers rather than lack of an effect.

## Conclusion

We have shown that pCR is less likely in tumours with mammographic spiculation. Furthermore, smaller ultrasound size is a positive predictor of response to NACT in HER-2 positive tumours. These findings may be important in assisting decision-making regarding offering breast cancer patients NACT.

## Data Availability

The datasets used and/or analysed during the current study are available from the corresponding author on reasonable request.

## Abbreviations

AUC: Area under the curve
DCIS: Ductal carcinoma in situ
ER: Oestrogen receptor
HER-2: Herceptin-2
MMG: Mammogram
MRI: Magnetic resonance imaging
NACT: Neoadjuvant chemotherapy
pCR: Pathological complete response
PET-CT: Positron emission tomography – computed tomography
PR: Progesterone receptor
ROC: Region under the curve
TN: Triple negative
US: Ultrasound
